# Editorial

**Published:** 1983-10

**Authors:** 


### EDITORIAL

With this number our Centenary Year ends. It has shown that our revival can be sustained. A wealth of good material has been supplied to the editor and it is with great pleasure that he thanks, on behalf of our readers, all those who have contributed. Especially our thanks are due to our panel of regular Correspondents who have come up regularly with information and comment and have made their section the most popular item in the *Journal*. In this number, amongst other contributions, Dr. Whitfield discusses Computers in General Practice, Dr. Tribe discusses staffing imbalances and Dr. Mather muses on 'Holistic and Alternative Medicine'. Of course the treatment of the whole patient is exactly what General Practice has always stood for in this country and Alternative Medicine is simply those modes of therapy which have failed to impress a profession always on the look out for advance – even for novelty. *Plus ça change*, etc. Dr. Thomson has paid a generous tribute to the Salvation Army who brighten the hospital Sunday with the 'sound of the cornet, the sackbut and all kinds of music'.

Bristol can still produce a good *Journal* but our income must be supplemented by all means available – of which advertising is the most satisfactory for all concerned. Members of the Society can help – see inside front cover.

